# Teacher Discrimination Reduces School Performance of African American Youth: Role of Gender

**DOI:** 10.3390/brainsci8100183

**Published:** 2018-09-30

**Authors:** Shervin Assari, Cleopatra Howard Caldwell

**Affiliations:** 1 Department of Psychiatry, University of Michigan, Ann Arbor, MI 48109, USA; 2 Center for Research on Ethnicity, Culture and Health, University of Michigan School of Public Health, Ann Arbor, MI 48109, USA; cleoc@umich.edu; 3 Department of Psychology, University of California Los Angeles (UCLA), Los Angeles, CA 90095, USA; 4 Department of Health Behavior and Health Education, University of Michigan School of Public Health, Ann Arbor, MI 48109, USA

**Keywords:** population groups, ethnic groups, race, ethnicity, racism, discrimination, Blacks, African Americans, gender, bias, teacher discrimination

## Abstract

Background: Gender may alter African Americans’ vulnerability to discrimination. The type of outcomes that follow exposure to discrimination may also be gender-specific. Although teacher discrimination is known to deteriorate school performance, it is yet unknown whether male and female African American youth differ in the effect of teacher discrimination on school performance. Objective: This cross-sectional study explored the moderating role of gender on the effect of teacher discrimination on school performance in a national sample of African American youth. Methods: The National Survey of American Life-Adolescent Supplement (NSAL-A) enrolled a nationally representative sample (*n* = 810) of 13–17-year-old African American youth. Demographic factors, socioeconomic status, teacher discrimination, and school performance (grade point average, GPA) were measured. Linear multivariable regression models were applied for data analysis. Results: Males and females reported similar levels of perceived teacher discrimination. In the pooled sample, higher teacher discrimination was associated with lower school performance among African American youth (*b* = −0.35; 95% confidence interval (CI) = −0.49 to −0.22). Gender interacted with perceived teacher discrimination (*b* = 12; 95% CI = 0.24–2.02), suggesting a significant difference between males and females in the magnitude of the association between perceived teacher discrimination and GPA. In stratified models, perceived teacher discrimination was associated with worse school performance of females (*b* = −12; 95% CI = −0.03 to −2.78) but not males (*b* = 0.01; 95% CI = −0.07 to 0.08). Conclusion: In line with previous studies, gender was found to alter the vulnerability of African American youth to perceived discrimination. African American boys and girls may differ in their sensitivity to the effects of teacher discrimination on school performance.

## 1. Introduction

Perceived racial discrimination is a common experience across US institutions and systems [1,2,3,4], and is a main contributor to the racial and ethnic gap in well-being of Americans [5,6,7,8,9,10,11,12,13]. Perceived discrimination is a well-established risk factor for multiple physical and mental health outcomes [14,15,16,17], and evokes a wide range of undesired outcomes [9,18,19,20,21,22,23,24,25,26,27]. Perceived discrimination is linked to negative emotions [18] such as depression, anxiety, and distress [18,19,20,21], behavioral risk factors such as suicide [23], substance use [9,24,25,26,27], and obesity [28], hyper-vigilance [29], and social isolation [22]. Perceived teacher discrimination (PTD) is also a predictor of poor school performance defined as low grade point average (GPA) [30,31] and school dropout [32,33].

Although all age groups of African Americans report perceived discrimination [29,34,35,36,37,38], African American youth who start developing their social and racial identity may perceive more discrimination [36,37,38]. Among African Americans, gender affects both perception as well as sensitivity to discrimination [30,31]. Minority males report more discrimination than minority females, a pattern which is shown in African American and other ethnic groups [27,36,39,40,41]. Gender also alters the harmful effects of discrimination [19,42,43,44], with females being more prone to the effects of discrimination on obesity and eating disorders [45] and males being more prone to the effects of discrimination on psychological distress [21], anxiety/depression [20], and substance use [46]. Thus, exposure and sensitivity to discrimination are not merely shaped by race and ethnicity, but by the intersection of gender and race/ethnicity [21,23,30,31,42,44,47]. As each intersectional social group (e.g., race/ethnicity by gender groups) has a unique life history, values systems, expectations, support systems, attributions, and norms [48], causes and consequences of the very same discriminatory experiences may vary for them. African American males and females are not an exception in this regard. Similar patterns are also shown for other types of environmental stressors [19,42].

Although PTD is an important type of discrimination against African American youth [49], very limited knowledge exists regarding gender differences in the effects of PTD on school performance of African American youth [30,31]. While overall, youth with PTD report poor school performance [50], this effect may differ for boys and girls [30,31]. However, available studies have mostly used local samples [30,31], leaving a gap in the knowledge that is generalizable to the US population. As a result, there is a need for studies that recruit a nationally representative sample of adolescents. In addition, there is a need for studies with large sample sizes, which generate statistical power to test moderations and mediations of PTD on youth outcomes (e.g., school performance). 

To fill the gap on the effects of PTD on school performance among African American youth, we explored gender variation in the link between PTD and grade point average (GPA) in a national sample of African American youth. In line with the past literature on male–female differences in exposure and sensitivity to discrimination [30,31,47,51] and other types of stress [19,42], we expected an interaction between gender and discrimination. We expected a larger effect of PTD on GPA in males compared to females. 

## 2. Methods

### 2.1. Design and Setting

This cross-sectional study used data from the National Survey of American Life-Adolescent Supplement (NSAL-A) [36,52]—a landmark national mental health survey of African American youth [53], conducted as part of the Collaborative Psychiatric Epidemiology Surveys (CPES) [54]. The NSAL-A is one of the very few available studies that makes a distinction between ethnic groups of Blacks. The NSAL (CPES) was conducted by the Institute for Social Research (ISR) at the University of Michigan (UM).

### 2.2. Ethical Considerations

The NSAL-A study protocol was approved (B03-00004038-R1) in the year 2003 by the University of Michigan (UM) Institutional Review Board (IRB). Youth provided assent. Youth legal guardians signed written informed consent. 

### 2.3. Participants and Sampling

The analytical sample in the current study was 810 African American adolescents aged 13 to 17 years old. Detailed description of the NSAL sampling and methodology is published elsewhere [55]. Participants were limited to African American youth who resided in US territories at the time of survey. The NSAL-A study measured the race/ethnicity of the youth according to the self-assigned race/ethnicity of the adolescent’s household. Parents who lived in the same households as youth self-identified themselves as African American, being defined as Blacks with no ancestral ties to any Caribbean countries.

The NSAL adolescent sample was drawn from the same households as the NSAL adult samples. The NSAL used a national probability sample of African American households that resided in the US. The NSAL households were screened for eligible youth who were living in the same household. Households with adolescents were randomly selected. When multiple eligible youth were living in the household, two adolescents were enrolled based on their gender.

### 2.4. Interviews and Data Collection

Interviews were all conducted in English, and took approximately 100 min. The response rate of the NSAL-A was 80.5% for African American youth. About 82% and 18% of the interviews were in-person and by phone, respectively. Face-to-face interviews were conducted in the adolescents’ homes. In-person interviews used computer-assisted personal interviews (CAPIs). CAPI-trained interviewers used computers to conduct interviews. CAPI is a preferred interview method when the questionnaires are complex and long [56].

### 2.5. Measures and Variables

Demographic information: The study included two demographic variables, namely age and gender. While age was operationalized as a continuous measure, ranging from 13 to 17, gender was treated as a dichotomous factor (female as the reference category).

Family income: The only socioeconomic status (SES) indicator in this study was family income. Family income was asked from the parent and was imputed for missing data. Higher family income was indicative of higher SES.

Perceived teacher discrimination (PTD): The NSAL-A used the following three items to measure PTD. Questions focused on experiences that occurred during the past year in the interaction with teachers at school. Similar to the Everyday Discrimination Scale (EDS) [57], items assess chronic, subtle, routine, and daily discrimination (in contrast to major and overt events) [15,58,59]. The items were: (1) “Your teachers treat you with less respect than other students”, (2) “Your teachers act as if they think you are not smart”, and (3) “Your teachers act as if they are afraid of you”. The response item for each question was on a six-level Likert scale ranging from 1 (never) to 6 (almost every day). A total score was calculated to reflect the frequency of PTD during the past year, ranging from 3 to 18, with a higher score indicating more discrimination by the teachers (α = 0.86).

GPA: The following single item was used to measure GPA—“What kind of grades (do/did) you usually get?” Responses were on a five-item response scale, including (1) Failing Grades; (2) Ds; (3) Cs; (4) Bs; and (5) As. GPA was operationalized as a continuous measure. A higher score reflected a better school performance.

### 2.6. Statistical Analysis

To adjust for the complex design of the NSAL-A, Stata 15.0 (Stata Corp., College Station, TX, USA) was used for data analysis. The NSAL-A sample lacks independence. Data were weighted to adjust for non-independence as well as the selection probabilities and non-response at the household and individual levels [15]. Taylor expansion approximation was used to re-calculate the design-based variance. Standard errors (SEs) reflect the study weights due to the complex sampling design. We reported frequency and mean (SEs). Missing data were imputed for family income. Sub-pop survey linear regression models were applied for multivariable analyses. In the linear regression models, self-reported grade-point-average was the outcome variable, PTD was the predictor variable, and age, gender, and family income were the covariates. In the first step, the association of interest was calculated in the overall sample of African American youth (*Model 1*). In the next step, the gender by PTD interaction term was added to the model (*Model 2*). At the last step, gender-specific models were run for females and males (*Model 3* and *Model 4*). Unstandardized regression coefficients (*b*), their 95% confidence intervals (CIs), and *p*-values were reported. *p*-values smaller than 0.05 were considered significant. 

## 3. Results

### 3.1. Descriptive Statistics

Table 1 provides a summary of the description of participating African American youth. Male and female African American youth did not differ in PTD. African American males reported lower GPA than African American females (Table 1).

### 3.2. Linear Multivariable Regression Models

Table 2 shows the results of two linear multivariable regression models in the overall sample of African American youth, with PTD as the predictor variable, GPA as the outcome variable, and gender, age, and family income as the covariates. *Model 1* only entered the main effects. *Model 2*, however, also included the gender by PTD interaction term.

In the overall sample of African American youth, PTD was associated with lower GPA (*b* = −0.35; 95% CI = −0.49 to −0.22). Gender interacted with PTD (*b* = 12; 95% CI = 0.24–2.02), suggesting a significant difference in the magnitude of the association between PTD and GPA between male and female youth (Table 2).

### 3.3. Stratified Linear Regression Models

Table 3 shows a summary of the results for *Model 3* and *Model 4* which were estimated in females and males, respectively. In stratified models, PTD was associated with school performance for females (*b* = −12; 95% CI = −0.03 to −2.78) but not males (*b* = 0.01; 95% CI = −0.07 to 0.08). (Table 3).

## 4. Discussion

In a national sample of African American youth, the current study showed three major findings: First, males and females reported similar levels of PTD. Second, more PTD was associated with worse GPA among all youth. Third, PTD showed a larger effect on the school performance of females than males.

The findings were unexpected and different from previous studies that have shown stronger effects of discrimination on various outcomes for males than for females. Not many studies have found that girls are harmed to a greater extent by perceived discrimination than boys. Such differences may be in part due to the differences in context, design, sample, sampling, and other methodological aspects across studies.

Very few studies have compared boys and girls for the effect of discrimination on school outcomes of African American youth. In a unique study by Chavous et al. [31], 204 male and 206 female African American adolescents in Grades 8 and 11 were examined. The study showed gender differences in the impact of earlier and later peer discrimination experiences on academic outcomes. For boys but not girls, racial centrality was associated with better school performance. Additionally, boys and girls differed in the moderating effect of racial centrality on the relationship between discrimination and academic outcomes. For boys, higher racial centrality diminished the risk for lower school grades due to experiencing classroom discrimination. For girls, higher racial centrality was protective against the negative impact of peer discrimination on academic constructs. For girls, peer discrimination related positively to academic self-concept in the presence of low race-centrality. Girls and boys also differed in how SES moderated the relationship between perceived discrimination and academic outcomes. The authors suggested that interactions between individual- and contextual-level factors are needed to better understand the role of discrimination on the academic and social development of African American youths [31].

In our study, compared to boys, girls’ school performance showed a larger negative effect due to teacher discrimination. Theories of gender socialization might help us to understand why gender may matter in the effects of social distress and social undermining on various developmental outcomes (e.g., PTD on school performance). Gender is a social construct, and shapes socialization and the differential impact of problematic social relations [60]. 

The higher sensitivity of girls’ GPA to PTD may be in part because girls’ school performance is better overall (more room for change due to PTD) [61]. Teachers’ expectations are also higher for females than males [62], and thus any decline of these expectations may be more consequential for girls than boys. Another explanation is that girls more commonly engage and rely on the exchange of social support with other members of their social network [63,64,65]. Thus, poor quality of relationship with teachers (due to discrimination) may be more damaging to girls than to boys. The same is shown for supportive relations with parents in prior studies [66]. 

Social relations have differential effects on the socialization and outcomes of girls and boys [67]. The greater sensitivity of females compared to males to PTD is in line with research showing stronger impact of change in social relations on females than males [68]. However, the results are in contrast to previous studies that suggest males may be more sensitive to discrimination and other types of environmental stress on outcomes such as distress, depression, and substance use [15,19,21,29,69].

A considerable body of literature suggests that gender [21], gender norms [41,48], socioeconomic status [26,69,70,71,72,73], racial identity [9,38,41,43,74,75], and racial attribution [43] change exposure and sensitivity to perceived discrimination. How ambiguous situations are interpreted depend on a wide range of traits and factors such as racial identity that shape the salience of race in day-by-day encounters [27,74]. Masculine ideologies may explain some of the observed findings regarding male gender sensitivity to discrimination [41,48,59,75]. Gender norms that shape beliefs about dominance and hierarchy may make males vulnerable to discrimination [59,75,76]. Males and females also differ in coping strategies and styles [41,77]. While males more frequently use confrontational coping [78], females more commonly use avoidant coping [77]. Compared to females, males more commonly act out their stress and emotions [79,80]. Males also do not use social supports in the same way as females [81].

African American families have major roles in shaping gender differences in sensitivity to discrimination [82,83]. African American parents differently deliver race socialization messages to their sons and daughters [82,83]. The effects of these messages may not be similar for boys and girls [84]. Male and female youth may also differ in peers’ influences [85]. Messages from different sources may ultimately result in a wide range of gender differences in expectation and response to discrimination across settings including schools. Gender differences in socialization may contribute to gender-specific effects of discrimination. While African American parents often tend to provide more race-related messages to their sons than to their daughters [86,87], they should also recognize that their daughters are very vulnerable to PTD.

The finding that male and female African American youth report similar PTD was unexpected. Some theories and empirical reports suggest that males report high exposure to discrimination. The Subordinate Male Target hypothesis suggests that across all ethnic groups, males are subject to more discrimination than females [30]. Thus, social patterning of discrimination not only depends on race/ethnicity, but on the intersection of gender and race/ethnicity as well [88,89]. Thus, it is not the addition of social identities but the multiplication of them that is associated with exposure to discrimination, which is in line with the intersectionality framework [90,91,92,93].

Due to the racial profiling of African American males, African American males experience high levels of threat-based discrimination. Similar findings are reported for other ethnic groups, such as Arab Americans [21], Latinos [44], and Caribbean Blacks [46].

Gender should be regarded as a central construct for studying the effects of racism on child development [35]. In a cross-sectional study among Caribbean Black youth, perceived discrimination was more common among males with darker skin tones. However, skin color did not influence perceived discrimination among females [94]. In other longitudinal studies, male gender was a risk factor for an increase in perceived discrimination over an 18-year follow-up period [88,89]. 

While research has consistently documented discrimination in racial and ethnic minority populations, far less is known about specific sources of discrimination such as those in various settings and enacted by various perpetrators. In this study, we focused on a particular type of discrimination—teacher discrimination. In our study, African American males and females reported similar levels of discrimination by teachers. However, it was female not male youth who were sensitive to the effects of teacher discrimination on school performance. A large body of evidence shows gendered response to discrimination, but the outcomes that follow discrimination are specific to gender [21,23,30,31,42,44,47]. 

Studies suggest that among various groups of racial and ethnic minorities, males are at a disadvantage in terms of exposure to discrimination as well as effects of discrimination on depression, distress, and substance use [19,27,36,39,40,41,42,43,44,88,89]. Despite existing policies [95], African American males and females are commonly target of discrimination by institutions such as the educational system, police, the correctional system, as well as the labor market [95]. Teachers also commonly discriminate against African American male and female students [30,31].

Although we did not find a main effect of gender on PTD, the media portrays African American males and females differently. That is, the media shapes different stereotypes against African American boys and girls in the minds of Americans, including teachers. The US media portrays African American males as aggressive and anti-intellectual [40,41,96,97] and African American females as sexual [98]. African American males have been stereotyped as “endangered, aggressive, angry, superhuman, subhuman, lazy, hyperactive, jailed, and paroled, on probation, lost, loveless, incorrigible, or just simply self-destructive” [99,100]. These stereotypes particularly evoke discrimination against African Americans [94].

Among African Americans, discrimination and other environmental stressors may have gender-specific effects, with females developing obesity and eating disorders [45] while males develop substance use and depression [44,96,97,101]. Discrimination has stronger effects on psychological distress [21], depressive and anxiety symptoms [19,20], and major depressive disorder (MDD) [101] for males than females. This study extends the current knowledge by showing that females may be more sensitive to the effects of PTD on school performance.

### 4.1. Limitations

The current study is not free from methodological limitations. First, the data were old—the data were collected 15 years ago. Still, the NSAL-A is one of the very few nationally representative data sets that are available on African American youth. This study failed to include relevant measures such as attribution, vigilance, personality, identity, and coping that may explain gender differences in the effect of discrimination on various outcomes. We also did not include contextual data from school (e.g., % whites and Blacks at school). The study also failed to collect data on teachers (age, gender, race). Such data could help in clarifying the discrimination pattern. In addition, we did not have any information on the type of school or socioeconomic status of school. The validity of self-reported GPA is well-documented. As a result, despite the limitation in the outcome measurement, this paper still makes a unique contribution to the literature, given the established validity of the outcome variable. Despite these limitations, recruiting a nationally representative sample of African American youth with a large sample size should be regarded as a strength of this study. Our findings contribute to the literature on gender differences in child and academic development [61,102,103,104] among African Americans.

### 4.2. Implications

All types of discrimination are inhumane [105], as they devalue individuals and diminish peoples’ sense of dignity and pride [106,107]. On top of such profound effects on self-esteem [108], perceived discrimination has other detrimental effects on a wide range of undesired and negative effects on health, well-being, and development [5,6,7,29,34,35,36,37,38]. There are already multiple legislations, laws, and regulations that ban discrimination in all settings [95]. The main problem is not a lack of laws, but that they are not enforced enough [109]. Instead of new policies, there is a need to reinforce existing laws.

Although schools are one of the main contexts for adolescent development [110], they are not the only locations where African American youth are discriminated against. Teacher discrimination should be seen in line with several other systems and institutions that unjustly treat African Americans. African American males disproportionately become targets of police brutality, mass incarceration, and stop and frisk policies and practices [111,112,113,114]. Systems such as school, police, and correctional settings need to introduce new trainings in their system to reduce blunt reactions toward African American youth, particularly those with larger body sizes and darker skin colors [94]. The same may apply to schools, teachers, and principals, who can discriminate against African American youth [115,116,117]. Teacher discrimination may increase the risk of drop out and suspension [118,119,120,121], as well as school performance.

Several measures and interventions can be used to decrease discrimination by school authorities. Increasing the diversity of the teacher pool is one step. White teachers should receive training to reduce implicit and explicit bias against African American students. Schools should receive a specific budget to educate school personnel on bias, racism, prejudice, and discrimination. Schools may make diversity a priority. Zero tolerance for discrimination may also be a step toward reducing the unfair treatment of African American students. Other initiatives with the potential to reduce teacher discrimination should be also explored.

### 4.3. Future Research

There is a need for studies that decompose actual and enacted discrimination from those which are merely perceived. Research needs to study how gender shapes attribution, vigilance, and identity related to race and ethnicity. There is also a need to study how the development of gender norms shapes experiences of discrimination due to other social identities. Future research should also test how gender is a determinant of actual discrimination by teachers against African American youth. Additional research that uses longitudinal data with multiple observations is needed. Finally, studies should explore whether males’ increased sensitivity to discrimination is due to long-term chronic exposure to discrimination that alters vigilance, racial identity, and coping, or is actually enacted by teachers and principals.

## 5. Conclusions

Similar to the previous studies on gender differences in exposure and vulnerability to discrimination [19,27,36,39,40,41,42,43,44], the current study revealed gender differences in the effects of teacher discrimination on GPA among African American youth. Being generalizable to the US population, the results suggest that among African American youth, teacher discrimination is common in both males and females, but such discrimination may be a more salient determinant of school performance for females than males. There is a need to reduce racism and discrimination for both genders and at all levels. Programs that reduce teacher discrimination at schools may differently impact the school performance of male and female African American youth. 

## Figures and Tables

**Table 1:** Descriptive statistics among African American youth by gender.

Characteristics	All		Males		Females	
	Mean	95% CI	Mean	95% CI	Mean	95% CI ^b^
Age (years) *	15.21	15.08–15.34	14.80	14.59–15.01	15.55	15.44–15.65
Income ($1000) *	0.58	−8.08 to 9.25	1.77	−7.23 to 10.77	−0.40	−8.92 to 8.11
Perceived Teacher Discrimination (PTD)	−0.01	−0.12 to 0.09	0.04	−0.10 to 0.19	−0.07	−0.19 to 0.05
Grade Point Average (GPA) *	3.68	3.59–3.77	3.50	3.39–3.61	3.86	3.78–3.94

* *p* < 0.05 for comparison of males and females; ^b^ CI: confidence interval.

**Table 2:** Linear multivariable regression models in the overall sample of African American youth.

Characteristics	*b*	SE	95% CI		*t*	*p*	*b*	SE	95% CI		*t*	*p*
	*Model 1* (Main Effects)						*Model 1* (Interaction)					
Gender (Male)	−0.35 ***	0.07	−0.49	−0.22	−5.36	0.000	−0.12 *	0.04	−0.21	−0.03	−2.74	0.011
Perceived Teacher Discrimination (PTD)	−0.05 *	0.02	−0.10	0.00	−2.18	0.038	0.02	0.02	−0.03	0.06	0.77	0.447
Age	0.02	0.02	−0.02	0.06	0.88	0.388	0.00	0.00	0.00	0.00	1.69	0.101
Income	0.00	0.00	0.00	0.00	1.64	0.113	−0.35 ***	0.06	−0.48	−0.22	−5.44	0.000
Gender (Male) * Perceived Teacher Discrimination (PTD)	-	-	-	-	-	-	0.12 *	0.06	0.00	0.24	2.02	0.050
Intercept	3.58 ***	0.31	2.95	4.21	11.63	0.000	3.61 ***	0.30	2.99	4.24	11.86	0.000

Outcome—grade point average (GPA); *b*—regression coefficient; * *p* < 0.05; ** *p* < 0.01; *** *p* < 0.001.

**Table 3:** Stratified linear regression models in female and male African American youth.

Characteristics	*b*	SE	95% CI		*t*	*p*	*b*	SE	95% CI		*t*	*p*
	*Model 3 (Females)*						*Model 4 (Males)*					
Perceived Teacher Discrimination	−0.12 **	0.04	−0.21	−0.03	−2.78	0.010	0.01	0.04	−0.07	0.08	0.16	0.875
Age	0.03	0.03	−0.03	0.09	1.05	0.301	0.00	0.02	−0.05	0.05	0.08	0.934
Income	0.00	0.00	0.00	0.00	0.97	0.340	0.00	0.00	0.00	0.00	1.13	0.268
Intercept	3.40 ***	0.42	2.54	4.26	8.12	0.000	3.47 ***	0.33	2.79	4.16	10.38	0.000

Outcome—grade point average (GPA); *b*—regression coefficient; * *p* < 0.05; ** *p* < 0.01; *** *p* < 0.001.
